# The Risk Factors for Radiation Pneumonitis after Single-Fraction Carbon-Ion Radiotherapy for Lung Cancer or Metastasis

**DOI:** 10.3390/cancers13133229

**Published:** 2021-06-28

**Authors:** Takashi Ono, Naoyoshi Yamamoto, Akihiro Nomoto, Mio Nakajima, Yuma Iwai, Yuka Isozaki, Goro Kasuya, Hitoshi Ishikawa, Kenji Nemoto, Hiroshi Tsuji

**Affiliations:** 1 Department of Radiation Oncology, QST Hospital, Chiba 263-8555, Japan; yamamoto.naoyoshi@qst.go.jp (N.Y.); nomoto.akihiro@qst.go.jp (A.N.); nakajima.mio@qst.go.jp (M.N.); iwai.yuma@qst.go.jp (Y.I.); isozaki.yuka@qst.go.jp (Y.I.); kasuya.goro@qst.go.jp (G.K.); ishikawa.hitoshi@qst.go.jp (H.I.); tsuji.hiroshi@qst.go.jp (H.T.); 2 Department of Radiation Oncology, Faculty of Medicine, Yamagata University, 2-2-2, Iida-Nishi, Yamagata 990-9585, Japan; knemoto@ymail.plala.or.jp

**Keywords:** heavy ion radiotherapy, radiation pneumonitis, dose fractionation, radiation, pulmonary neoplasms, lung diseases, interstitial

## Abstract

**Simple Summary:**

There was no reports about the risk factors of high dose single-fraction carbon-ion radiotherapy. Although there were only small number of patients with symptomatic radiation pneumonitis after this treatment, we showed that the risk factors of radiation pneumonitis include the dose–volume parameter.

**Abstract:**

There are no studies on the risk factors of radiation pneumonitis (RP) after carbon-ion radiotherapy at a dose of 50 Gy (relative biological effectiveness (RBE)) in a single fraction. The objective of this study was to identify factors associated with RP after radiotherapy, including dose–volume parameters. Ninety-eight patients without a history of thoracic radiotherapy who underwent treatment for solitary lung tumors between July 2013 and April 2016 were retrospectively analyzed. Treatment was planned using Xio-N. The median follow-up duration was 53 months, and the median clinical target volume was 32.3 mL. Three patients developed grade 2 RP, and one patient developed grade 3 interstitial pneumonitis. None of the patients developed grade 4 or 5 RP. The dose-volume parameters of the normal lung irradiated at least with 5–30 Gy (RBE), and the mean lung dose was significantly lower in patients with grade 0–1 RP than in those with grade 2–3 RP. Pretreatment with higher SP-D and interstitial pneumonitis were significant factors for the occurrence of symptomatic RP. The present study showed a certain standard for single-fraction carbon-ion radiotherapy that does not increase the risk of RP; however, further validation studies are needed.

## 1. Introduction

In 2018, 2.1 million new lung cancer cases were detected, accounting for approximately 12% of all new cancer cases, along with a high mortality rate, accounting for approximately one in five (18.4%) cancer-related deaths. The incidence and mortality rates in men with lung cancer were the highest in both developed and developing countries in 2018 [1]. Surgery is the first choice of treatment for early-stage lung cancer [2].

However, many patients are medically contraindicated to undergo surgical resection or refuse to undergo surgery because of poor performance status, older age, and complications. In such patients, stereotactic body radiotherapy (SBRT) is recommended as a radical treatment [2]. Various dose fractionation schedules, including single-fraction treatment, are effective with tolerable toxicities [3,4,5]. Moreover, a recent study based on the National Cancer Database revealed that SBRT is associated with improved overall survival compared to that with no treatment in patients with early-stage non-small cell lung cancer (hazard ratio: 0.56) [6]. Therefore, SBRT is an important treatment choice for patients with lung cancer, especially for inoperable patients.

Radiation pneumonitis (RP) is a frequent and problematic toxicity associated with SBRT. RP can be occasionally fatal. Therefore, some patients discontinued SBRT due to a high risk of RP. Various studies have revealed risk factors associated with the development of RP, including dose–volume histograms [7,8,9]. Those studies have reported that a lower ratio of the irradiated area is associated with lower incidence of RP.

Particle therapy is also a treatment choice for lung cancer, and proton beam therapy (PBT) and carbon-ion radiotherapy (CIRT) are primarily used worldwide. Regarding the treatment schedule followed worldwide, PBT requires multiple fraction doses [10,11]. In contrast, patients with lung cancer have been treated with CIRT since 1994 at our institution [12], and we gradually reduce the dose fractionation [13,14]. Patients with primary lung cancer or lung metastasis from a primary lung cancer are treated with CIRT at a dose of 50 Gy (relative biological effectiveness (RBE)) in a single fraction after a dose escalation study [15]. We have previously reported the clinical effectiveness of CIRT at a 50 Gy (RBE) single-fraction dose for non-small cell lung cancer [16].

CIRT has the advantage of reducing the irradiation dose to normal tissue due to increased energy deposition with a penetration depth up to a sharp maximum at the end of its range, also known as the Bragg peak [17]. Moreover, CIRT is known to reduce the lung doses more than SBRT or PBT [18,19].

However, there are no studies detailing the risk factors for RP in patients who have received 50 Gy (RBE) single-fraction CIRT. Therefore, this study aimed to identify risk factors associated with RP after CIRT, including the dose–volume parameters.

## 2. Materials and Methods

### 2.1. Ethics Statement

This study was approved by the institutional ethics committee of the QST hospital (approval number: 19-035). The study was conducted in accordance with the principles of the Declaration of Helsinki.

### 2.2. Patients

Patients who received CIRT for primary lung cancer or lung metastasis from lung cancer between July 2013 and April 2016 at QST hospital were identified and retrospectively analyzed. The stage of primary lung cancer was determined according to the Union for International Cancer Control (7th edition) [20] and findings of computed tomography (CT), positron emission tomography-CT, and brain magnetic resonance imaging. Patients with the following criteria were included in the study: the patient received 50 Gy (RBE) single-fraction passive CIRT, the treatment was planned using Xio-N, and the patient had a solitary lung tumor, World Health Organization performance status of 0–2, no lymph node metastasis, and no distant metastasis to other organs or other sites of uncontrolled cancer. Patients who had received concurrent chemotherapy and prior irradiation to the lungs were excluded. Patients with interstitial pneumonitis (IP) were also included in the present study. Patients were diagnosed with IP according to their medical history, respiratory function test results, and abnormalities in the lungs on chest CT, such as reticular opacities.

### 2.3. Carbon-Ion Radiotherapy

Treatment planning for CIRT was based on three-dimensional CT images acquired at 2.5 or 5.0 mm intervals during the exhalation phase. All patients were immobilized in the supine or prone position using Moldcare (Alcare, Tokyo, Japan) or Shelfitter (Kuraray, Osaka, Japan). CIRT was planned using Xio-N (ELEKTA, Stockholm, Sweden; Mitsubishi Electric, Tokyo, Japan). The gross tumor volume (GTV) included lung tumors, and the clinical target volume (CTV) included the GTV plus a margin of 0.5–1.0 cm. Spicule formation and pleural indentation were also included in the CTV. The planning target volume (PTV) included the CTV plus the internal margin, which considered respiratory movements of each patient’s tumor. The patients were rotated by a maximum of ±20 °C with three to four different angle ports between the horizontal and vertical beams. Carbon-ion beams of 250, 350, or 400 MeV, depending on the target size and water equivalent path length along the beamline, were generated using the Heavy Ion Medical Accelerator in Chiba. The total dose was applied to the isocenter and tuned to accommodate the PTV with a 95% isodose line of the prescribed dose. The CIRT field was positioned based on digitally reconstructed radiographs, X-ray imaging, and metal markers made of iridium wire as landmarks. The CIRT dose is shown in Gy (RBE), calculated by multiplying the physical dose with the RBE [21].

### 2.4. Evaluation and Follow-Up

Patients were followed up every 1–3 months in the first year and every 3–6 months thereafter; most patients underwent CT to assess for RP after CIRT. RP was evaluated using the Common Terminology Criteria for Adverse Events version 4.0 [22].

### 2.5. Statistical Analysis

Statistical analyses were performed using IBM SPSS Statistics software (version 24, SPSS Inc., Chicago, IL, USA). The mean values of the dose–volume parameters and pretreatment clinical factors, except IP, were compared using the Mann–Whitney *U* test. The dose–volume parameters included the ratio of lung volume irradiated with at least x Gy (RBE) (Vx) (V5, V10, V15, V20, V25, and V30) and mean lung dose (MLD). The pretreatment clinical factors included the percentage of predicted forced expiratory volume in 1 s, the percentage of predicted vital capacity, and the expression of Krebs von den Lungen 6 (KL-6) and surfactant protein D (SP-D). The relationship between IP and RP occurrence was compared using the chi-square test. Receiver operating characteristic (ROC) curves and sensitivity/specificity calculations were performed to decide the cut-off value of the dose–volume parameter. Then, the chi-square test was performed to evaluate them. All *p*-values were two-sided, and *p*-values < 0.05 were considered statistically significant. Multivariate analysis was not performed due to the smaller number of events.

## 3. Results

### 3.1. Patients

We identified 104 patients with lung tumors who received single-fraction passive CIRT (50 Gy (RBE)) between June 2013 and April 2016. Six patients were excluded because of a history of thoracic radiotherapy received before single-fraction CIRT. Finally, 98 patients met the inclusion criteria, including seven (7.1%) patients with lung metastases (Table 1). Single-fraction CIRT was successfully administered in all the patients. The median lung tumor diameter was 23.0 mm (range, 7.5–49.0 mm). The median follow-up duration was 53 months (range, 5–79 months). Fifteen (15.3%) patients had IP, including three patients who received treatment for IP. All patients with IP had primary lung cancer and were inoperable.

### 3.2. Radiation Pneumonitis

**Figure 1:**
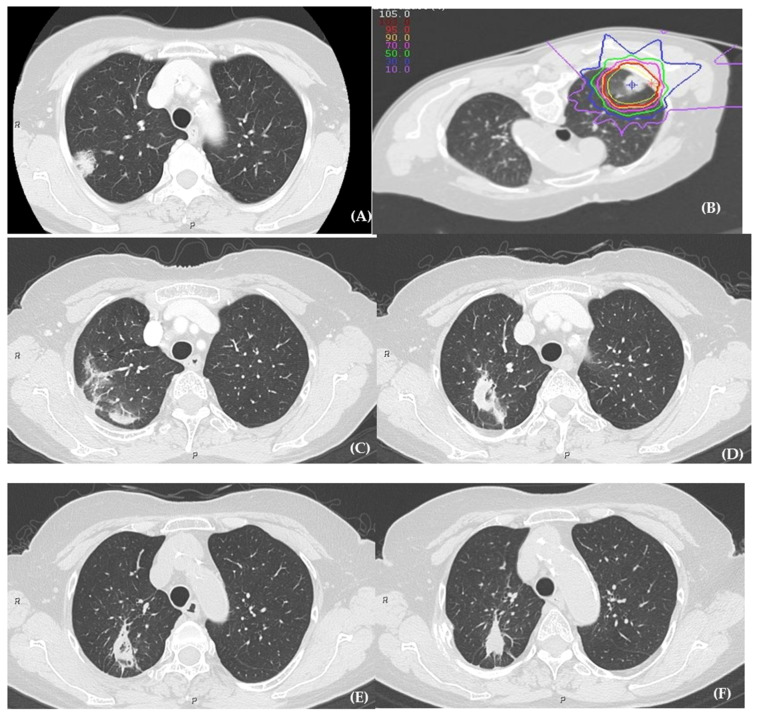
The course of grade 1 radiation pneumonitis after carbon-ion radiotherapy. (**A**) The pretreatment lung tumor. (**B**) The treatment plan for the patient. The patient received carbon-ion radiotherapy in prone position with four ports. (**C**) The lung image 6 months after treatment. The infiltration shadow is spread around the irradiated area of the lung. (**D**) The lung image 1 year after treatment. The infiltration shadow gradually shrunk. (**E**) The lung image 3 years after treatment. (**F**) The lung image 7 years after treatment. The infiltration shadow shrunk, and there is no evidence of local recurrence.

Figure 1 shows a typical course of grade 1 RP after CIRT. The infiltration shadows generally spread widely around the irradiated area several months after CIRT. Further, it gradually shrunk over 1–3 years if there were no local recurrences. Grade 2 RP was observed in three patients after 4–6 months of administering single-fraction CIRT (two patients without IP and one patient with IP), and they received steroid administration. One patient with IP developed grade 3 RP 3 months after CIRT. The patient was treated with steroid pulse therapy and oxygen therapy. IP treatment before the CIRT was not administered in patients with symptomatic RP. None of the patients developed grade 4 or 5 RP.

**Figure 2:**
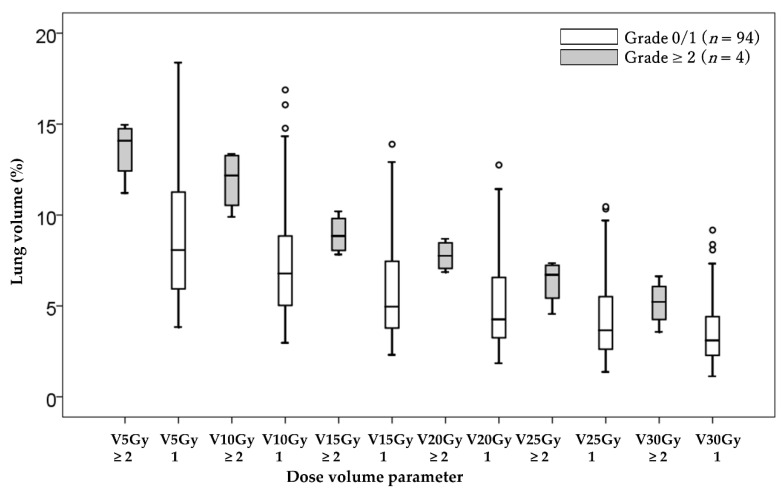
Comparison of the dose–volume parameter of all lungs between patients with grade 0–1 and grade 2–3 radiation pneumonitis. Abbreviation; Vx: normal lung volume irradiated at least xGy (relative biological effectiveness); RBE: relative biological effectiveness.

**Figure 3:**
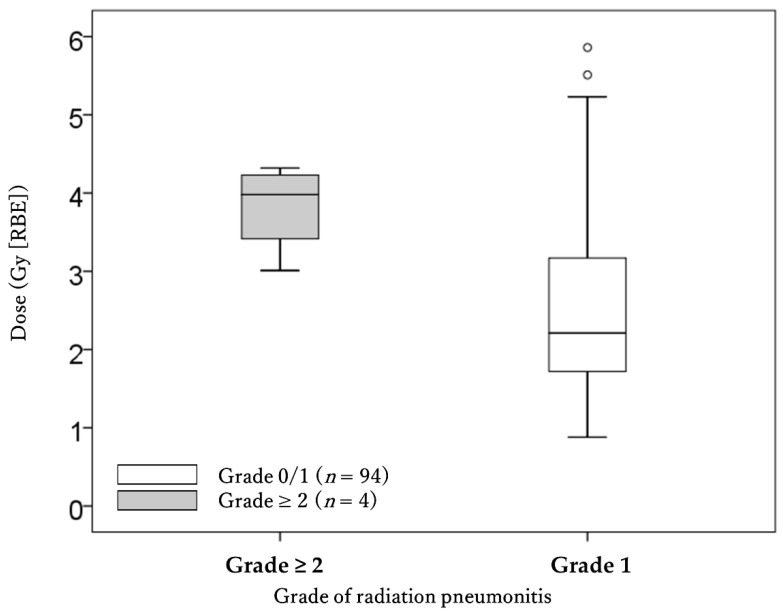
Comparison of the mean lung dose of all lungs between patients with grade 0–1 and grade 2–3 radiation pneumonitis. Abbreviation; RBE: relative biological effectiveness.

In the univariate analysis, the dose–volume parameters for V5-30 and MLD were significantly lower in patients with grade 0–1 RP than in those with grade 2–3 RP (Table 2, Figure 2 and Figure 3). The data were used to calculate ROC curves, and the cut-off values of dose parameters were V5 = 11.0% (sensitivity 100%, specificity 74.5%), V10 = 9.4% (sensitivity 100%, specificity 75.5%), V15 = 7.8% (sensitivity 100%, specificity 79.8%), V20 = 6.8% (sensitivity 100%, specificity 79.8%), V25 = 4.5% (sensitivity 100%, specificity 64.9%), V30 = 3.5% (sensitivity 100%, specificity 59.6%), and MLD = 3.0 Gy (RBE) (sensitivity 100%, specificity 71.3%). Then, patients who received an irradiated dose of more than those cut-off values had a significantly higher rate of grade 2–3 RP (Table 3). There was little irradiated area in the contralateral lung, and there were no significant differences in contralateral MLD between patients with grade 0–1 RP and those with grade 2–3 RP (0.053 Gy (RBE) vs. 0.056 Gy (RBE), *p* = 0.948).

### 3.3. Correlation between Pretreatment Clinical Factors and Radiation Pneumonitis

**Figure 4:**
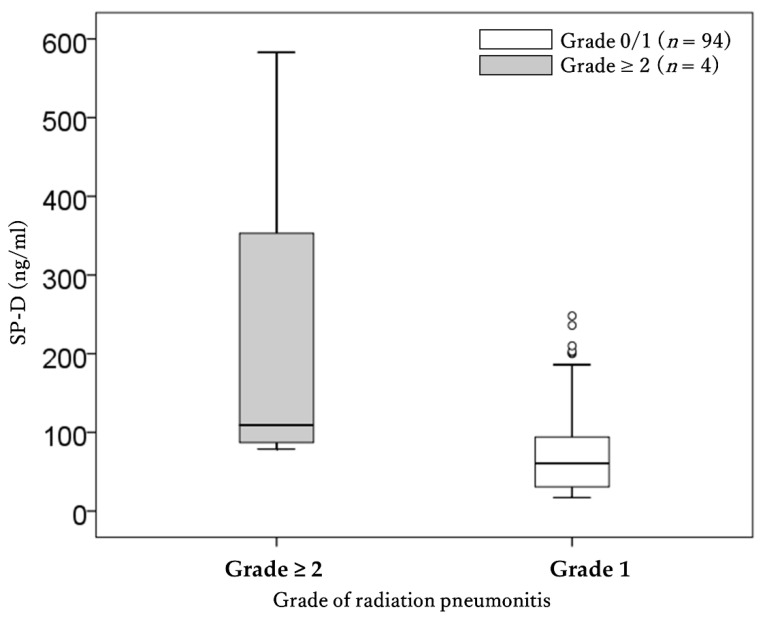
Comparison of pretreatment SP-D between patients with grade 0–1 and grade 2–3 radiation pneumonitis.

SP-D was a significant factor for the occurrence of grade 2–3 RP (Table 4 and Figure 4). IP was also a significant risk factor for the occurrence of grade 2 or 3 RP (13% vs. 2%, *p* = 0.049).

## 4. Discussion

To the best of our knowledge, this is the first study on the risk factors of RP after 50 Gy (RBE) single-fraction CIRT.

Previous studies on SBRT have reported that MLD and lung V5, V10, and V20 in patients without symptomatic RP were 2.7–4.0 Gy and 13.8–20%, 8.0–12%, and 2.6–4.0%, respectively; however, those of patients with symptomatic RP were 3.4–5.1 Gy and 18.4–27.0%, 11.4–16.3% and 3.5–6.8%, respectively [7,23,24]. In this study, the dose–volume parameter for V20 was equal to or slightly higher than that in previous studies. This may be because the CTV in the present study was larger than that in previous studies, as the margins were included in the GTV in CIRT planning. In contrast, the CTV was equal to the GTV in most of the SBRT plans. However, V5 and V10 in the present study were much lower than those reported in previous studies. These low-dose parameters were attributed to the Bragg peaks of the carbon ions. Ebara et al. reported that treatment with CIRT can reduce the irradiation dose to the lung more than that with SBRT, particularly in the low-dose areas [18]. Barriger et al. reported that an MLD > 4 Gy was a risk factor for the incidence of symptomatic RP after SBRT [7]. Here, the cut-off MLD values for symptomatic RP were lower than those in the study by Barriger et al. Thus, MLD > 4 Gy (RBE) may also be a risk factor for symptomatic RP after 50 Gy (RBE) single-fraction CIRT, although the dose calculation varied between CIRT and SBRT because of differences in the RBE.

Irradiation of the contralateral lung is a risk factor associated with symptomatic RP after chest irradiation. Bongers et al. reported that lower contralateral MLD was the only significant factor associated with grade ≥ 3 RP [25]. They reported that planning constraints should aim to keep the contralateral MLD < 3.6 Gy. In contrast, contralateral MLD in the present study was very low and was not a significant factor associated with symptomatic RP. As carbon ions increase energy deposition, with penetration depth reaching a sharp maximum at the end of its range, few beams reached the contralateral lung in CIRT for lung cancer. This may be a reason for the lower number of patients with symptomatic RP after CIRT in the present study than that in other studies.

IP is one of the most severe problems in the treatment of lung cancer. A systematic review by Chen et al. reported that the treatment-associated mortality in patients who received SBRT was 15.6%, which was significantly higher than that in patients who received particle therapy (4.3%) [26]. Onishi et al. analyzed multi-institutional data in Japan and reported a large population correlation between fatal RP and IP; the rate of occurrence of fatal RP was 6.9% [27]. A lower number of patients was included in the present study, but none of them developed fatal RP. One probable explanation is that CIRT for lung tumors can reduce MLD. In a study by Chen et al. MLD ≤ 4.5 Gy was associated with reduced mortality [26]; they also reported that particle therapy reduced treatment-related mortality more than that with SBRT (4.3% vs. 15.6%). Moreover, Kim et al. compared PBT and X-ray therapy directly by using retrospective data and concluded that PBT may be more helpful in reducing fatal complications than X-ray therapy [28], although there have been no reports comparing CIRT and X-ray therapy. Particle therapy, including CIRT, administered in patients with IP may reduce post-treatment lung toxicities more than SBRT. In contrast, Yamashita et al. reported that high SP-D and KL-6 levels were risk factors for severe RP after SBRT [29]. However, the present study had less events, and only SP-D was a risk factor for symptomatic RP.

The beam arrangement for lung tumors is important in CIRT. In the treatment of peripheral lung tumors, the beam arrangement is simpler, as we can easily choose the short carbon-ion pathway. However, the dose–volume parameters can be several, depending on the beam angles because of the long carbon-ion pathway, if the tumors are near the center of the lung. Moreover, lung tissue has low stopping power due to low material density, and it takes an approximately 3–4-fold longer distance for carbon-ion to stop than water-equivalent tissues [30,31]. Therefore, we should reduce the number of carbon-ion beam angles to minimize the lung volume involved in beam pathways; this may lead to a lower occurrence of RP. Furthermore, reducing the total number of ports may also lead to a lower irradiated lung volume. We are currently evaluating the utility of this method, and it will be reported soon.

This study had some limitations. First, it was a retrospective study performed at a single institution. Second, the number of patients with symptomatic RP was very low because of the low number of patients. Therefore, a multivariate analysis could not be performed. In fact, the number of patients could be increased if we included patients who received scanning CIRT; however, due to differences in planning for scanning CIRT and passive CIRT, we could not include those patients in the present study. We believe that the dose–volume parameter of scanning CIRT should be evaluated separately from that of the passive CIRT. Third, this study included patients with IP, which itself was a risk factor for RP after CIRT. Therefore, further validation studies are needed, especially for patients with IP.

## 5. Conclusions

The present study showed a certain cut-off standard for 50 Gy (RBE) single-fraction CIRT that does not lead symptomatic RP. The following standard dose–volume parameters were identified: V5 = 11.0%, V10 = 9.4%, V15 = 7.8%,V20 = 6.8%, V25 = 4.5%, V30 = 3.5%, and MLD = 3.0 Gy (RBE). However, the number of patients with symptomatic RP was very low because of the single-institution study, and further studies are needed to validate our findings.

## Figures and Tables

**Table 1:** The patient characteristics (*n* = 98).

Characteristics	Patients
Age (years)	
Median (range)	76 (42–95)
Gender	
Male	68 (69.4%)
Female	30 (30.6%)
Performance status	
0	76 (77.6%)
1	22 (22.4%)
Operable or inoperable	
Operable	46 (46.9%)
Inoperable	52 (53.1%)
Follow-up time (months)	
Median (range)	53 (5–79)
Tumor location	
Right upper lobe	28 (28.6%)
Right middle lobe	6 (6.1%)
Right lower lobe	27 (27.6%)
Left upper lobe	23 (23.5%)
Left lower lobe	14 (14.3%)
Histopathology	
Adenocarcinoma	31 (31.6%)
Squamous cell carcinoma	13 (13.3%)
Non-small cell lung cancer	1 (1.0%)
Clinical lung cancer	46 (46.9%)
Metastasis from lung cancer	7 (7.1%)
Interstitial pneumonitis	
Yes	15 (15.3%)
No	83 (84.7%)
Diameter of lung tumor (mm)	
Median (range)	23.0 (7.5–49.0)
Volume of clinical target volume (ml)	
Median (range)	32.3 (7.9–137.5)

**Table 2:** The correlation between dose–volume parameters of all lung and radiation pneumonitis.

Parameter	Grade1 (mean ± SD)	Grade2/3 (mean ± SD)	*p*
V5	8.7 ± 3.3%	13.6 ± 1.7%	0.006 *
V10	7.4 ± 3.1%	11.9 ± 1.7%	0.007 *
V15	5.7 ± 2.6%	8.9 ± 1.1%	0.009 *
V20	5.0 ± 2.3%	7.8 ± 0.8%	0.011 *
V25	4.2 ± 2.1%	6.3 ± 1.3%	0.028 *
V30	3.6 ± 1.8%	5.2 ± 1.3%	0.047 *
MLD	2.5 ± 1.1 Gy (RBE)	3.8 ± 0.6 Gy (RBE)	0.014 *

Abbreviation; SD: standard deviation; Vx: normal lung volume irradiated at least x Gy (relative biological effectiveness); MLD: mean lung dose; RBE: relative biological effectiveness.*; *p* < 0.05.

**Table 3:** The comparison before and after cut-off values calculated by using receiver operating characteristic curves.

Parameter	Comparison	Number of Patients	Ratio of Grade 2–3 RP	*p*
V5	≤11.0%	70	0	0.006 *
	>11.0%	28	14%	
V10	≤9.4%	71	0	0.005 *
	>9.4%	27	15%	
V15	≤7.8%	74	0	0.003 *
	>7.8%	24	17%	
V20	≤6.8%	74	0	0.003 *
	>6.8%	24	17%	
V25	≤4.5%	61	0	0.018 *
	>4.5%	37	11%	
V30	≤3.5%	58	0	0.025 *
	>3.5%	40	10%	
MLD	≤3.0 Gy (RBE)	67	0	0.009 *
	>3.0 Gy (RBE)	31	13%	

Abbreviation; RP: radiation pneumonitis; Vx: normal lung volume irradiated at least xGy (relative biological effectiveness); MLD: mean lung dose; RBE: relative biological effectiveness.*; *p* < 0.05.

**Table 4:** The correlation between pretreatment clinical factors and radiation pneumonitis.

Parameter	Grade1 (Mean ± SD)	Grade2/3 (Mean ± SD)	*p*
%FEV 1.0	80.6 ± 20.5%	70.7 ± 23.7%	0.332
%VC	97.1 ± 18.9%	86.8 ± 23.9%	0.389
KL-6	329.9 ± 236.0 U/mL	495.4 ± 319.6 U/mL	0.250
SP-D	80.9 ± 75.9 ng/mL	220.1 ± 242.7 ng/mL	0.026 *

Abbreviation; SD: standard deviation; %FEV1.0: percent predicted forced expiratory volume in one second; %VC: percent predicted vital capacity. *; *p* < 0.05.

## Data Availability

Data sharing is not applicable to this article.
